# Association of Trace Elements with Polycystic Ovary Syndrome in Women—A Case-Control Study

**DOI:** 10.3390/metabo15020079

**Published:** 2025-01-29

**Authors:** Tinkara Srnovršnik, Bojana Pinter, Milena Horvat, Janja Snoj Tratnik, Ingrid Falnoga, Darja Mazej, Ivan Verdenik, Irma Virant-Klun

**Affiliations:** 1 Divison for Women’s Healthcare-Šiška Unit, Community Health Centre Ljubljana, Metelkova ulica 9, 1000 Ljubljana, Slovenia; tinkara.srnovrsnik@zd-lj.si; 2 Faculty of Medicine, University of Ljubljana, Vrazov Trg 2, 1000 Ljubljana, Slovenia; bojana.pinter@guest.arnes.si; 3 Division of Obstetrics and Gynecology, University Medical Centre Ljubljana, Šlajmerjeva 3, 1000 Ljubljana, Slovenia; ivan.verdenik@guest.arnes.si; 4 Department of Environmental Sciences, Jožef Stefan Institute (JSI), Jamova cesta 39, 1000 Ljubljana, Slovenia; milena.horvat@ijs.si (M.H.); janja.tratnik@ijs.si (J.S.T.); ingrid.falnoga@ijs.si (I.F.); darja.mazej@ijs.si (D.M.); 5 Clinical Research Centre, University Medical Centre Ljubljana, Vrazov Trg 1, 1000 Ljubljana, Slovenia

**Keywords:** essential and non-essential elements, PCOS, diet, habits, oxidative stress, endocrine disruptors, hepatic damage, renal toxicity

## Abstract

**Objectives:** There are still limited or lacking data on the association of trace elements (TEs) with polycystic ovary syndrome (PCOS). This case–control study aimed to determine levels of essential TEs (manganese (Mn), copper (Cu), zinc (Zn), selenium (Se), molybdenum (Mo)) and non-essential TEs (arsenic (As), cadmium (Cd), mercury (Hg), lead (Pb)) in urine, whole blood, and serum to investigate a possible association with kidney and liver function, endocrine and metabolic parameters, and environmental and lifestyle sources of potential exposure and provide possible recommendations. **Methods:** In our case–control study, women with PCOS (*n* = 35) and healthy controls (*n* = 35) underwent clinical and ultrasonographic examination, filled in questionnaires targeting general, lifestyle, and environmental information, and provided fasting venous blood samples and first morning urine for biochemical, hormonal, and TE analysis. Multiple linear regression models were used to evaluate the association between TE levels and data obtained through questionnaires. **Results:** In women with PCOS, lower Mo levels in whole blood (*p* = 0.024) and serum (*p* = 0.011) and higher serum Cu levels (*p* = 0.026) were detected when compared to healthy controls. Results of this study show that amendments in Cu and Mo levels might be related to altered kidney and liver function and disrupted hormonal balance in PCOS women. Cu levels positively correlated with leukocyte count. There was a negative correlation of Mo levels with proteinuria and luteinizing hormone levels. Regarding liver function, Mo negatively correlated with urinary bilirubin levels, and there was a positive association with alanine and aspartate aminotransferase, respectively. Dietary supplement consumption and certain diet habits appeared to be important predictors of exposure to Cu (beef consumption) or Mo (cereal and boiled vegetable consumption) and modify Mo and Cu levels in women. **Conclusions:** Concentrations of the chemical elements Mo and Cu in biological samples of women appear to be related to PCOS and nutrition. To our knowledge, this is a novel finding for Mo. Additional research is needed to provide more insights into the causality of the PCOS relationship with Mo and Cu in humans.

## 1. Introduction

Polycystic ovary syndrome (PCOS) is the most common endocrine disorder, affecting 5–10% women of reproductive age [1]. It can be diagnosed if two out of the following three criteria are fulfilled: (1) clinical and/or biochemical hyperandrogenism, (2) chronic oligomenorrhea and/or anovulation, and/or (3) the presence of polycystic ovaries on transvaginal ultrasonography [2]. PCOS is associated with metabolic complications, e.g., obesity, dyslipidaemia and insulin resistance (IR) [3]. A substantial heterogeneity of the disorder reflects its still enigmatic pathophysiologic and etiologic background. Therefore, PCOS is regarded as a complex multifactorial disorder resulting from genetic, environmental, and lifestyle interactions [4]. Growing evidence suggests that oxidative stress (OS) may also play a role in the pathogenesis of PCOS by inducing IR and hyperandrogenism [5,6].

The serum levels of trace elements (TEs)—essential and non-essential TEs—may be different in patients with PCOS [7]. TEs are found abundantly in the environment and human exposure can occur through environmental or occupational interaction, as well as through accumulation in food and water [8]. Numerous non-essential TEs such as arsenic (As), mercury (Hg), cadmium (Cd), and lead (Pb), have been shown to disrupt various body systems, and chronic exposure to these TEs can lead to cumulative damage, including OS, cellular toxicity, and DNA damage [4,5]. They can disrupt endocrine function by interfering with reproductive hormone regulation and can potentially contribute to metabolic imbalances such as diabetes and increased body mass index (BMI) and waist circumference [4]. Essential elements, such as selenium (Se), molybdenum (Mo), manganese (Mn), copper (Cu), and zinc (Zn) are components (cofactors) of metalloenzymes or coenzymes that regulate various enzymatic reactions related to reproduction, e.g., ovulation, metabolism, and hormone management [9,10]. Evidence suggests the association of altered levels of essential elements with metabolic syndrome and PCOS [5]. Therefore, several TEs have been shown to exert effects similar to metabolic and hormonal dysfunction seen in PCOS.

Both essential and non-essential TEs can be related to human reproduction. Cd has been associated with higher levels of oestradiol [11] and decreased follicle-stimulating hormone (FSH) levels in serum [12]. It may also contribute to OS, interfere with the DNA repair process, and induce hyperglycaemia through various mechanisms [4,7]. Pb might exert antiestrogenic effects [11] and play a role in the pathogenesis of PCOS by enhancing lipid peroxidation [13]. It remains unclear by which mechanisms As exerts toxic effects on the female reproductive system [14]. As-induced OS can lead to the apoptosis of pancreatic β-cells and a decrease in insulin secretion [15]. Hg can lead to increased lipid peroxidation and OS, therefore causing menstrual abnormalities and low follicle count [5,16]. Studies evaluating the association of Cd, Pb, As, and Hg blood levels with endocrine and metabolic parameters in PCOS are scarce with conflicting results [4,17,18,19,20].

Se constitutes active centres of selenoproteins. It exhibits antioxidant and anti-inflammatory effects [21,22]. It acts antagonistically to non-essential TEs such as As, Cd, Pb, and Hg [23]. Decreased plasma Se levels have been linked to hyperandrogenism in PCOS [21], and Se supplementation showed beneficial effects in PCOS women by improving reproductive and inflammatory markers [24]. Cu is a cofactor of many enzymes involved in redox reactions alone or in association with zinc (Zn) [25,26]. On the contrary, its excess or deficiency can also induce OS and cause alterations in sex hormone activity [26,27,28]. Higher serum Cu levels were found in PCOS women with insulin resistance (IR) [26]. Zn is involved in androgen and insulin metabolism [29]. Although not entirely consistent, many studies showed elevated serum Cu levels and lower Zn levels in PCOS women [3,17,29,30,31]. Mn is a component of the mitochondrial superoxide dismutase (SOD) antioxidant system [26]. Recent studies revealed increased serum Mn levels in PCOS women [31,32], which is in contrast to previous research [7,26]. To our knowledge, there are currently no studies available on the possible association of Mo with PCOS. In the development of PCOS, multiple mechanisms including OS, low-grade inflammation, and impaired antioxidant defence have been implicated as contributory factors [33]. Mo can exert both pro-oxidative and antioxidative properties and has been established as an insulin-mimetic compound [34]. It forms an active site of molybdoenzymes, which metabolize a variety of endogenous and exogenous compounds involved in the pathogenesis of obesity, insulin resistance, or diabetes, which are all features commonly seen in women with PCOS [34].

The presence of TEs in the human body, including both essential and non-essential elements, may among other effects, impact reproductive health. TEs are found ubiquitously in the environment, resulting in daily individual exposure and accumulation over time. Given the exposure to various TEs simultaneously, the complex interactions and potential synergistic and antagonistic effects among these TEs highlight the need to examine their combined influence. Considering limited or lacking data on the association of selected TEs with PCOS phenotype, the present study aimed to (1) determine the levels of TEs (Mn, Cu, Zn, As, Se, Mo, Cd, Hg, Pb) in different body components—urine (U), whole blood (WB), and serum (S)—in the PCOS and control groups of women to conduct a more thorough analysis and provide a more accurate assessment of TE metabolism, storage, and excretion; (2) investigate a possible association with kidney and liver function and endocrine and metabolic parameters due to a lack of scientific research; (3) evaluate a possible association with environmental and lifestyle habits in order to identify sources of potential acute or chronic exposure; and (4) consequently provide the basis to establish future recommendations to minimize the harmful effects of selected TEs on female reproductive health. 

## 2. Materials and Methods

### 2.1. Study Population

This retrospective case–control study was conducted between October 2022 and July 2023 at the Division of Obstetrics and Gynecology, University Medical Centre Ljubljana. Informed written consent was obtained from all participants. The participants were able to withdraw from the study at any time. The study was conducted in accordance with the Declaration of Helsinki and was approved by the Republic of Slovenia National Medical Ethics Committee (protocol code 0120-530/2021/6, 15 February 2022).

The study included 35 women diagnosed with PCOS (study group) and 35 healthy women (control group) aged 20–39 years. The recruitment of 35 participants per group aligns with sample sizes from comparable studies, yielding meaningful results and significant associations [7,20,31]. To maintain a more homogeneous study population and to minimize variability related to weight and adiposity that could obscure our findings, only women with the body mass index (BMI) range of 18.5–29.9 kg/m^2^ were included in the study. The diagnosis of PCOS was based on the Rotterdam criteria with the presence of two out of the following three features: oligo- or anovulation, clinical and/or biochemical signs of hyperandrogenism, and/or polycystic ovary morphology (presence of ≥12 follicles 2–9 mm in diameter in at least one ovary) on ultrasound [35]. Women who were admitted for routine control, with a menstrual cycle duration of 21–35 days, and who did not meet the PCOS diagnostic criteria were included in the control group. The exclusion criteria were pregnancy, hormonal treatment in the last three months (hormonal contraception, hormone replacement therapy, progestogens), hyperprolactinemia, thyroid dysfunction, premature ovarian failure, primary amenorrhea, endometriosis, and chronic diseases such as diabetes, depression, Crohn’s disease, celiac disease, cardiovascular diseases, liver diseases, kidney diseases, asthma, chronic obstructive pulmonary disease, musculoskeletal disorders, neurological disorders, and oncological diseases.

Clinical and transvaginal ultrasonographic evaluations of endometrial thickness and anthral follicle count (AFC) were carried out with a 2.0–11.0 MHz vaginal transducer (Samsung WS80A ultrasound system). The calculation of ovarian volume (OV) was performed using the simplified formula for a prolate ellipsoid (0.5 × length (cm) × width (cm) × thickness (cm)). Greater abdominal or visceral adiposity is associated with greater IR, which could exacerbate the reproductive and metabolic disorders in PCOS [35]. In addition, the presence of IR and its compensatory hyperinsulinemia is associated with increased bone mass, which could be detected by a simple measurement of the WC [36,37]. Therefore, the wrist circumference (WC) was recorded.

### 2.2. Questionnaire Data

Following clinical and ultrasonographic examination, women filled in questionnaires targeting (1) general and lifestyle information (age, geographical region of birth and residence, ethnicity, education level and occupation, body weight and height, menstrual cycle characteristics, gynecological medical history, smoking status, coffee and alcohol consumption, dental amalgam fillings, and metal body implant exposure), (2) food consumption and nutritional habits (dietary supplement consumption, type of water supply, daily water intake), food container materials (frequencies of storage in plastic, ceramic, metal, glass, PVC, paper, tetrapak), food reheating in containers, frequency of use and age of sports bottles (PVC, metal), frequency of consumption for various types of food (dairy products, fats, fruit and vegetables, meat and eggs, carbohydrate-rich foods, condiments, snacks, and beverages), (3) living environment (age of residence, type of heating, indoor environment characteristics, vicinity of manufactural industry (less than 1 km away) and waste disposal sites (less than 3 km away), agriculture), and (4) use of personal care products (lipstick, cream, hair balsam, hair styling products, perfume, deodorant, body oil, and nail polish), pest control agents, and plant protection products. The questionnaires were validated in previous national biomonitoring [38].

### 2.3. Sample Analysis

#### 2.3.1. Biochemical and Hormonal Assays

All biological samples were handled under the patient code to protect personal data. Venous blood samples and first morning urine were collected after an overnight fast between the second and the fifth day of the spontaneous menstrual cycle or anytime following ≥ 2 months of amenorrhea at the Institute of Clinical Chemistry and Biochemistry, University Medical Centre Ljubljana. A hemogram, serum tests such as aspartate aminotransferase (AST), alanine aminotransferase (ALT), gamma-glutamyl transferase (γ-GT), creatinine, cholesterol, high-density lipoprotein-cholesterol (HDL), low-density lipoprotein–cholesterol (LDL), triglycerides, 25-hydroxycholecalciferol (25(OH)D_3_)), and urine tests for liver and kidney function (specific gravity (SG), pH, glucose, hemoglobin, creatinine, albumin, the albumin/creatinine ratio, bilirubin, urobilinogen, methyl ketones, the IgG/creatinine ratio, alpha1-microglobulin/creatinine ratio) were performed by using standard routine in-house methods for diagnostics in daily clinical practice. In addition, the levels of sex hormone-binding globulin (SHBG) and reproductive hormones such as follicle-stimulating hormone (FSH), luteinizing hormone (LH), prolactin (PRL), thyroid-stimulating hormone (TSH), 17-beta oestradiol, progesterone, total testosterone (TT), androstenedione, and dehydroepiandrosterone sulphate (DHEAS) were determined.

#### 2.3.2. Analysis of Trace Elements

Urine was collected in 100 mL precleaned containers, and an additional 6 mL of venous blood was taken under sterile conditions in specialty tubes for the determination of TEs (Mn, Cu, Zn, As, Se, Mo, Cd, Hg, Pb). The biological samples were immediately aliquoted into 2 mL cryovials and stored long-term at −80 °C at the Clinical Research Centre, University Medical Centre Ljubljana. Biological samples were transferred on dry ice to the Department of Environmental Sciences at Jožef Stefan Institute for further analysis of TEs. TE analysis was performed in whole blood, serum, and urine samples by validated analytical methods. The samples were previously digested using a microwave digestion system (Ultrawave, Single Reaction Chamber Microwave Digestion System, Milestone). Measurements of Mn, Zn, Cu, Mo, Se, Pb, As, and Cd in digested samples were conducted using inductively coupled plasma mass spectrometry (ICP-QQQ, Agilent 8800) equipped with an octapole reaction system (ORS). All analyses were conducted under strict quality control protocols. Blank samples, control samples, and reference materials were analyzed alongside the test samples daily. The quality of the results was checked via the regular use of the reference materials Seronorm Trace Elements Whole Blood Level 1 and Level 2, Seronorm Trace Elements Serum Level 1 and Level 2, Seronorm Trace element Urine Level 1 and Level 2, (SERO, Billingstad, Norway), and also by participation in the German External Quality Assessment Scheme (G-EQUAS, Erlangen-Nuremberg, Germany).

To provide different insights into metal metabolism, storage, and excretion, the levels of TEs were analyzed in different body compartments, namely in urine (U), whole blood (WB), and serum (S), respectively.

The concentrations of TEs in urine were adjusted for variations in diuresis using specific gravity adjustment. In the context of environmental exposure to TEs, SG adjustment is less affected by body size, age, gender, muscle mass, or meat consumption and therefore appears to be a more reliable alternative compared to systematic creatinine adjustment [39]. As the standards in SG normalization calculations, SG means of 1.013 for females were used [38,40]. It has been observed that the Zn/Cu ratio could be a better marker for human metabolism compared to Zn or Cu status alone, and it seems to exert a significant effect on metabolism, indicating the pivotal role of these trace elements in the pathogenesis of OS and metabolic diseases [33,41]. Therefore, we separately calculated Zn/Cu ratios in urine, whole blood, and serum.

### 2.4. Statistical Analysis

The results were statistically analyzed using the IBM SPSS Statistics 29 package. Data distribution normality was determined using the Shapiro–Wilk test. The non-parametric Mann–Whitney U test was used to assess the differences in clinical characteristics and TE levels between the PCOS and control groups of women due to the abnormal distribution of data. The associations between TE levels and PCOS risk were adjusted for the confounding variables of age, BMI, and hematocrit (for whole blood levels of TEs, which are primarily bound to erythrocytes [42,43]). An ANOVA and Spearman’s correlation statistics were used to determine the correlation between different variables and among different TEs. Multiple linear regression models were used to evaluate the association between TE levels and data obtained through questionnaires. Statistical significance was set at *p* < 0.05.

The limits of TE detection (LODs) were LOD Mn = 0.40, LOD Cu = 0.40, LOD Zn = 40.0, LOD As = 0.10, LOD Se = 0.30, LOD Mo = 0.10, LOD Cd = 0.03, LOD Hg = 0.10, and LOD Pb = 0.20 ng/g. In whole blood, the respective LODs were LOD Mn = 1.10, LOD Cu = 0.40, LOD Zn = 25.0, LOD As = 0.03, LOD Se = 0.40, LOD Mo = 0.04, LOD Cd = 0.02, LOD Hg = 0.08, and LOD Pb = 0.10 ng/g. In serum, the respective LODs were LOD Mn = 0.30, LOD Cu = 0.80, LOD Zn = 120, LOD As = 0.07, LOD Se = 0.20, LOD Mo = 0.10, LOD Cd = 0.02, LOD Hg = 0.04, and LOD Pb = 0.09 ng/g. The data below the LOD were assigned values of LOD/$\sqrt{2}$.

## 3. Results

### 3.1. General Characteristics of the Study Population

The mean age in the PCOS and control groups of women was comparable, at 28.31 ± 5.09 and 28.80 ± 6.11 years, respectively. Regarding sociodemographic characteristics, there were no significant differences between the PCOS and control groups of women in the geographical region of residence and birth, education and employment status, vicinity of manufacturing industry and waste disposal sites, or agriculture. There were also no differences among both groups in coffee and alcohol consumption, as well as metal body implant exposure. Note that in the control group of women, there were marginally significantly more former smokers (Table 1).

However, women with PCOS reported a more frequent consumption of fresh fruit (*p* = 0.017), vitamin supplements (*p* = 0.005), and other types of dietary supplements (e.g., essential fatty acids or folic acid; *p* = 0.001), but they consumed less pasta when compared to the control group (*p* = 0.026). Differences in dietary patterns between the groups are specified in the Supplementary Materials (Table S1).

### 3.2. Anthropometric, Ultrasonographic, and Laboratory Features of the Study Population

Differences in anthropometric, ultrasonographic, and laboratory characteristics between the women in the PCOS and control groups are summarized in Table S2 in the Supplementary Materials. Statistically significant results are summarized in Table 2. Values are represented as mean ± standard deviation. Women with PCOS had higher WC and BMI measures (*p* = 0.027 and 0.004, respectively), a longer menstrual cycle length (*p* < 0.001), a thinner endometrium (*p* = 0.004), higher AFC levels (*p* < 0.001) and average ovarian volume (*p* < 0.001), lower serum FSH levels (*p* = 0.007), higher progesterone levels (*p* = 0.018), TT (total testosterone) levels (*p* = 0.006), androstenedione levels (*p* = 0.002), and FAI (free androgen index) levels (*p* < 0.001), as well as an increased leukocyte count (*p* = 0.048). Women with PCOS also more frequently reported on menstrual cycle irregularity (*p* < 0.001). There were no significant correlations observed between progesterone levels and endometrium thickness in the PCOS or control groups of women. In addition, there were also no differences observed between both study groups regarding lipid profiles and liver enzyme levels (Table S2 in the Supplementary Materials).

### 3.3. Trace Element Levels in Study Population

In women with PCOS, there were lower levels of Mo in whole blood ((Mo-WB), *p* = 0.024) and serum ((Mo-S), *p* = 0.011) and higher levels of Cu in serum ((Cu-S), *p* = 0.026) compared to the control group of women. SG-normalized Mo values (Mo-SG) in the PCOS group appeared to be marginally significantly lower (*p* = 0.052). However, these differences were no longer statistically significant after adjusting for age, BMI, and hematocrit. Table 3 and Table 4 summarize the concentrations of selected TEs in different biological samples, namely urine, whole blood, and serum, respectively.

#### 3.3.1. Sociodemographic Characteristics and Mo and Cu Levels

In both the PCOS and control groups of women, we found no significant correlations between the geographical region of birth and residence, education and employment status, workplace, coffee and alcohol consumption, metal body implant exposure, and Mo-SG, Mo-WB, Mo-S, and Cu-S levels. However, in the control group of women, there were associations with regard to smoking status. Non-smokers exposed to cigarette smoke had lower Mo-WB levels. In women with PCOS, we found some correlations with regard to residential environment characteristics. Living in the vicinity of waste disposal sites inversely correlated with Mo-WB (*p* = 0.013) and Mo-S levels (*p* = 0.002). The vicinity of fruit cultivation was negatively associated with Cu-S levels (*p* = 0.037). On the other hand, the vicinity of vegetable plots positively correlated with Mo-WB levels (*p* = 0.037).

#### 3.3.2. Nutritional Patterns, Laboratory Features, and Mo and Cu Levels

In the Spearman correlation analysis between selected TE levels (Mo-SG, Mo-WB, Mo-S, Cu-S), nutritional patterns, and laboratory features, we found some significant correlations in both groups of women.

**Figure 1:**
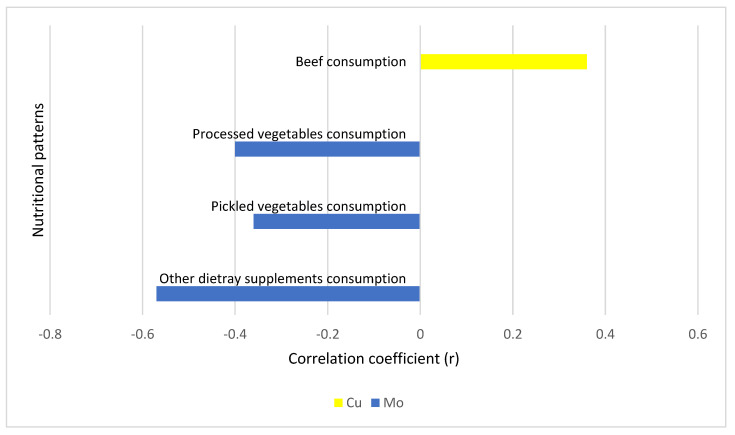
The correlations of Mo and Cu levels with nutritional patterns in women with PCOS (*n* = 35). Legend: Cu—copper; Mo—molybdenum. r—Spearman correlation coefficient; statistical significance: *p* < 0.05.

**Figure 2:**
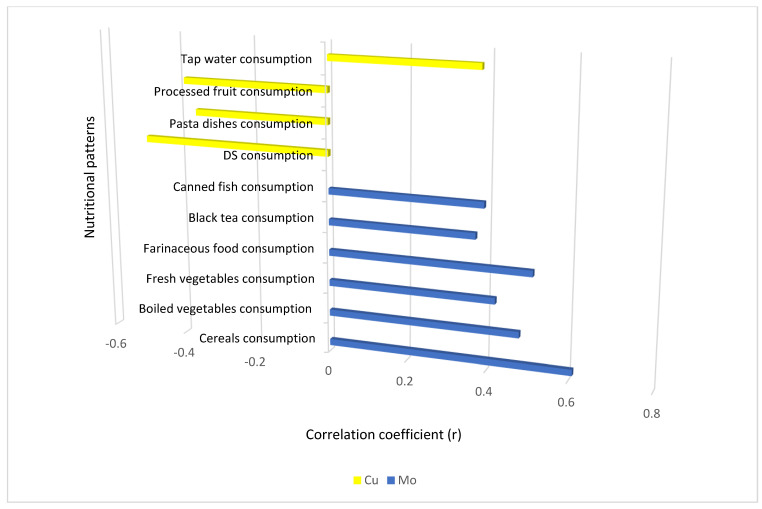
The correlations of Mo and Cu levels with nutritional patterns in the control group of women (*n* = 35). Legend: DS—other dietary supplements; Cu—copper; Mo—molybdenum. r—Spearman correlation coefficient; statistical significance: *p* < 0.05.

Mo and Cu concentrations in biological samples of women were related to their diet. Table 5 and Figure 1 and Figure 2 summarize statistically significant correlations between Mo and Cu levels and nutritional patterns in both groups. Regarding women with PCOS, Mo levels inversely correlated with pickled and processed vegetable consumption. In the control group of women, there were more significant associations between Cu, Mo, and nutritional habits detected when compared to women with PCOS. Cu levels negatively correlated to pasta dishes and processed fruit consumption and positively with tap water consumption. Mo levels showed some positive associations with the consumption of fresh vegetables, farinaceous food, black tea, and canned fish, but there were also negative associations with fruit juice consumption. Regarding food supplement consumption, the consumption of other types of dietary supplements was negatively correlated with Mo levels in women with PCOS and with Cu levels in the control group of women.

In addition, some other dietary habits also appeared to be associated with Mo and Cu levels in women. We found positive correlations of Cu values with beef consumption in women with PCOS and positive correlations of Mo levels with the consumption of cereals and boiled vegetables in the control group of women. The most important dietary sources associated with Mo and Cu levels in women are summarized in Table 6.

Mo and Cu contents in biological samples of women were also associated with their laboratory features. Statistically significant associations of Mo and Cu levels with laboratory findings in both groups are summarized in Table 7. When assessing hemogram parameters, there were positive correlations of Cu-S levels with leukocyte count and Mo levels with MCV and MCH in the PCOS group. On the other hand, there was an inverse correlation between Mo levels and MCV and MCH in the control group of women. Regarding hormonal features, we found a negative correlation between Mo and LH levels in women with PCOS. In the control group of women, there were positive correlations between Mo, TSH, and progesterone levels, while Cu-S was positively correlated with SHBG levels.

#### 3.3.3. Liver and Kidney Function and Mo and Cu Levels

Regarding liver function in women with PCOS, there were positive associations between blood Mo levels, AST, and ALT and negative associations with urinary bilirubin levels. Urinary urobilinogen was positively correlated with urinary Mo levels. When assessing kidney function, several correlations with Mo and Cu were revealed in both groups of women. In women with PCOS, Mo was negatively correlated with urinary SG, urinary protein levels, and urinary creatinine levels. In the control group of women, Mo was positively correlated with serum creatinine levels but negatively with U-pH. In both groups, the PCOS and control groups of women, Mo was positively correlated with urine SG and urinary creatinine levels.

#### 3.3.4. Zinc/Copper Ratios

Table S3 in the Supplementary Materials summarizes Zn/Cu ratios in biological samples in the PCOS and control groups of women. Differences in urine, whole blood, and serum between both study groups were not statistically significant.

In the control group of women, the Zn/Cu-WB ratio was negatively correlated with SHBG levels and urinary protein levels and positively with urinary SG and urinary creatinine levels.

### 3.4. Summary of the Most Important Results of the Study

Higher levels of Cu-S and lower levels of Mo-WB and Mo-S were observed in women with PCOS; urinary Mo levels (SG-normalized) were marginally significantly lower. Women with PCOS more often reported on vitamin and other types of dietary supplement consumption when compared to healthy controls. In women with PCOS, Cu-S levels positively correlated with beef consumption. In the control group of women, Mo levels were positively correlated with cereals and boiled vegetable consumption. The consumption of other types of dietary supplements was negatively correlated with Mo levels in women with PCOS and with Cu-S levels in the control group of women. In women with PCOS, Cu-S levels positively correlated with leukocyte count. Regarding hormonal characteristics, there was a negative correlation between Mo and LH levels in women with PCOS. In the control group of women, Mo was positively correlated with progesterone levels and Cu-S with SHBG levels, but there was a negative correlation of the Zn/Cu-WB ratio with SHBG levels. Regarding liver and kidney function, there were positive associations between Mo, AST, ALT, and urinary urobilinogen and negative associations of Mo with urinary bilirubin and protein levels in women with PCOS. In the control group of women, Mo was positively correlated with serum creatinine levels and negatively with U-pH. The Zn/Cu-WB ratio had a negative association with urinary protein levels.

## 4. Discussion

### 4.1. Comparison and Contrast with the Findings of Similar Studies

In this study, higher levels of Cu-S were observed in women with PCOS when compared to controls. In the literature, several other studies reported on elevated Cu levels in the serum of PCOS women within ranges similar to our results [7,17,20,25,26,28,29,31,32,46,47]. In addition, higher Cu levels in PCOS women were also found in their erythrocytes [10,48] and follicular fluid [49]. Cu exposure in humans occurs mainly through food intake. In the body, Cu is stored primarily in the liver and reaches the kidneys via circulation; excess of Cu is mainly eliminated in the feces [50] and is normally very low due to tight Cu regulation [51]. Whole blood Cu includes Cu bound to red blood cells and ceruloplasmin-bound Cu (90%), demonstrating both recent and long-term exposure [51]. Serum Cu is a good indicator of dietary intake and measures ceruloplasmin-bound Cu and loosely bound Cu, which is biologically active but tightly regulated by the body [51]. Elevated serum Cu levels may be seen in certain liver diseases, infections, and inflammation [51,52]. However, Cu levels obtained in our study were within the reference values for biological samples described in the literature for Slovenian and other populations [45], and its variability could also be related to lipid metabolism in the control and PCOS groups [28].

It has been speculated that the endocrinological and metabolic effects in PCOS may involve oxidative damage potentially mediated by Cu and other selected TEs [4,19,20,26,30,32,46,47]. In our study, we observed increased leukocyte count and a positive correlation of Cu-S with leukocyte count in women with PCOS, indicating a role of Cu in the OS response, although the underlying mechanisms remain unclear, and the possibility of this correlation indicating inflammation should also be considered. Conversely, in the control group, Cu levels appeared to interact with SHBG levels. This observation may suggest an impact of Cu on sex hormone regulation, as has been noted in the previous literature [53,54]. However, it is crucial to emphasize that Cu levels in both groups remained within the normal physiological range andwithout more compelling bioindicators from our study, attributing OS specifically to Cu remains speculative. OS in conditions like dyslipidemia often triggers cellular antioxidant responses, including the induction of protective buffer molecules and enzymes such as copper zinc superoxide dismutases (SOD1 and SOD3). These adaptive and homeostatic processes can sometimes be mistaken for causal etiologies. Given the limitations of our current data and the complexity of Cu’s role in metabolic pathways, including its involvement in lipid metabolism [55,56], we advise interpreting these findings with caution. Future studies with more robust indicators of OS and Cu metabolism will be necessary to clarify these relationships.

An important question is whether Cu content in biological samples is related to kidney function in women. In our study, a negative correlation of Zn/Cu-WB ratio with urinary protein levels in the control group of women could be suggestive of potential Cu-mediated renal damage, as observed in some studies on the general population [57,58,59].

Regarding Mo, this research represents a pioneering exploration of possible associations between Mo levels and PCOS. We observed lower levels of Mo-WB and Mo-S in the PCOS group compared to controls, with Mo-SG levels marginally significantly lower. These findings align with Mo’s physiological role as a cofactor for several metalloenzymes with oxidoreductase activity, including xanthine oxidase, which facilitates the conversion of tissue purines to uric acid [43,60]. Mo is primarily stored in the liver and kidneys and is excreted through urine [61]. Although Mo deficiencies are rare due to its trace occurrence in food [62], variations in Mo levels could reflect differences in dietary intake or increased utilization in metabolic processes. Importantly, all observed Mo concentrations remained within reference ranges, suggesting that lower levels in the PCOS group may point toward increased utilization rather than deficiency, potentially linked to heightened oxidative stress or depleted antioxidant mechanisms. This hypothesis is supported by recent insights into Mo enzyme functions, such as their roles in lipid metabolism and antioxidant regulation, which may influence reproductive health and metabolic homeostasis [61,63,64]. On the other hand, exposure to excess Mo levels in humans has been associated with adverse respiratory and renal effects, while scientific data on hepatic, uric acid level, and reproductive effects remain inconclusive [60]. Urinary Mo reflects recent environmental and occupational exposure to Mo [61,65]. Whole blood Mo levels indicate overall exposure and include Mo in the erythrocytes and plasma [43]. Serum Mo levels measure systemic exposure and demonstrate the nutritional status, metabolic impacts, and its interaction with other minerals like Cu. In animal models, excess Cu decreased Mo levels via reducing Mo absorption by forming non-absorbable Cu-Mo complexes in the gastrointestinal tract [66].

We found some significant correlations of Mo with regard to reproductive hormones. In women with PCOS, we observed a negative correlation between Mo-S and LH levels. Elevated LH and altered LH-to-FSH ratios are hallmark features of PCOS [2], suggesting a potential protective role for Mo in modulating gonadotropin levels. However, the exact mechanisms remain speculative, particularly given conflicting evidence from animal studies on Mo’s effects on reproductive health. [67,68,69,70,71,72,73]. In addition, Mo appeared to accumulate in several organs, e.g., pituitary or ovaries in a sheep model [74], leading to multiple pathological changes in the affected organs with a marked depletion of gonadotropin levels, suggesting the mechanism of a toxic endocrinopathy [75]. This is in line with some other animal models [76,77,78,79,80,81]. Data from human studies on Mo and LH levels are scarce, inconclusive, and limited to the male population [82,83]. Furthermore, in the control group of women, Mo-SG levels were positively correlated with progesterone. While this relationship is not well documented in the literature, it underscores the need for further research to elucidate Mo’s role in steroidogenesis and hormonal regulation, particularly during the follicular phase, when progesterone levels are typically stable.

Our study focused also on nutritional patterns and food supplements that are gaining popularity in recent years. According to significantly lower Mo levels and an inverse correlation with LH in women with PCOS, a potential protective role of Mo in reducing androgen levels in PCOS population can be speculated. Some studies have already proposed Mo as a new possible candidate medicine for the treatment of male [84] or female infertility [85]. There were women with PCOS that more often reported on vitamin and other food supplement consumption, presumably to improve their health status, but the question remains as to what extent the consumption of dietary supplements contributed to negative correlations with Mo-SG levels in women with PCOS and with Cu-S levels in the control group of women. The positive correlation of Cu-S levels with beef consumption, observed in women with PCOS, acknowledges that beef is an important dietary source of Cu [86]. It remains debatable as to what extent higher Cu intake might affect Mo metabolism by forming the aforementioned non-absorbable Cu-Mo complexes in the gastrointestinal tract [66]. Positive correlations of Mo levels with the consumption of cereals and boiled vegetables in the control group of women indicate that Mo-rich food might be a significant source of Mo [43]. We consider our findings not to be neglected for alleged future dietary recommendations and comprehensive content stratification of the commercially available dietary supplements in Slovenia and further large-scale research is mandatory to make a more proper distinction between different nutritional patterns and lifestyle habits in association with Mo and Cu levels in women, particularly in PCOS women.

Furthermore, we were also interested in the relationship between Mo and the possible impairment of kidney function. Although the Mo levels observed in our study are within reference ranges for a healthy population, suggesting no overt Mo-induced toxicity, statistical analysis revealed some associations worth discussing. An inverse correlation with proteinuria in women with PCOS and positive correlations with serum creatinine levels in the control group of women might reflect a potential negative impact of Mo on kidney function. Several animal models reported on Mo-induced nephrotoxicity via increased OS [87,88,89,90]. This is in line with several other studies in humans [91,92,93,94] but not with all of them [95]. However, it needs to be underlined that serum creatinine levels may also change due to non-renal factors independent of renal function such as muscle mass, nutritional status, and protein intake [96]. On the other hand, a recent study by Joun et al. carried out on a large sample pointed out the potential antioxidative benefits of Mo in human kidneys [97]. The authors also observed a reduced risk of hyperuricemia according to a negative correlation between urinary Mo levels and serum uric acid concentration. An inverse correlation between Mo levels and urinary pH detected in the control group of women could be potentially to some extent explained by increased uricosuria. This contradicts previous studies reporting that Mo overexposure contributes to the development of hyperuricemia and gout [98,99]. Therefore, we speculate whether significantly lower Mo levels, observed in the PCOS population in our study, could imply lower antioxidant capacity and a lack of the aforementioned renoprotective effect of Mo. On the other hand, the more often reported vitamin supplement consumption in women with PCOS raises a question as to whether these women might benefit from it in terms of decreasing serum uric acid. Data from in vitro and in vivo studies suggest that vitamin C reduces serum uric acid levels through several mechanisms, including by increasing urinary excretion [100]. However, it is not yet possible to assess the extent to which individual dietary habits might have an impact on Mo levels and kidney function in PCOS.

In addition, several significant associations with regard to Mo and liver function also merit some attention. A possible interference with hepatic function was indicated by positive correlations between urinary urobilinogen, AST and ALT levels, and Mo levels. Previous studies have speculated that Mo’s adverse effects on liver function may be due to increased OS, as seen in some animal models [101,102] and human studies [103]. In contrast, Kuang et al. found no significant association between Mo exposure and AST or ALT, suggesting instead that Mo exposure may more prominently affect renal rather than hepatic function, possibly mediating secondary hepatic effects [91]. On the other hand, emerging evidence also highlights Mo’s potential hepatoprotective properties, particularly its involvement in mitigating OS and lipid metabolism disorders. Recent studies have implicated mitochondrial amidoxime-reducing component (mARC), a Mo-dependent enzyme, in the pathogenesis of non-alcoholic fatty liver disease (NAFLD) and non-alcoholic steatohepatitis (NASH) [64]. These findings underscore the broader metabolic implications of Mo beyond its traditional enzymatic roles. In women with PCOS, a negative correlation of Mo with urinary bilirubin levels is also worth mentioning. If we assume that urinary bilirubin levels to some extent reflect blood bilirubin levels, our findings align with other studies [104,105]. This is noteworthy because bilirubin, known for its antioxidant properties [106], may also provide cardiovascular protection by exerting anti-lipemic, anti-inflammatory, and anti-diabetic properties [107]. The potential clinical relevance of altered Mo levels in association with liver function in PCOS should be thoroughly investigated in future studies.

In conclusion, while Mo levels in this study were within normal ranges, the observed correlations with reproductive hormones and its putative roles in OS and metabolic regulation warrant further investigation. Differences in Mo levels may reflect not only dietary intake but also increased metabolic demands or physiological stressors in PCOS. Future studies should explore these relationships using longitudinal designs and more extensive cohorts to clarify Mo’s functional significance in reproductive and metabolic health.

### 4.2. Limitations and Strengths of the Study

Certain limitations of our study merit attention. The present study was conducted on a relatively small sample size. On the other hand, women were included carefully and selectively based on their medical status. Our participant pool came almost exclusively from the urban Central Slovenia region, which should be considered when generalizing our findings to other more rural Slovenian regions. The weakness of our study is also a single sample collection and therefore laboratory features and TEs were measured at the same time, preventing us from determining the temporal association between exposure and outcome. In order to more properly evaluate kidney function, a 24 h urine collection would be more accurate. Since our study population was exposed to several TEs, it is in this context impossible to overlook the alleged co-exposure effects with other metals. On the other hand, the strength of our study is in providing a thorough overview of the differences in Cu and Mo levels in different biological matrices (urine, whole blood, and serum) and their potential clinical relevance. Our observations add a novel dimension to current evidence in understanding the pathophysiology of PCOS in relation to TEs, particularly Mo.

### 4.3. Implications of the Study Results for PCOS

We found some significant associations between Cu and Mo levels and certain nutritional habits and food supplement consumption. Given the importance of the antagonistic actions of Cu and Mo and the extremely rare Mo deficiency in humans, it remains debatable whether there the higher Cu levels in PCOS women are in fact a driving force contributing to clinical features in PCOS in the context of lifestyle and diet interactions. More research is needed to clarify this association with PCOS.

### 4.4. Suggestions for Future Research in the Field

Comprehensive, prospective, nationwide, multicentric studies are needed to further investigate and delineate if the alteration of dietary patterns and nutrient intake would lead to clinically meaningful outcomes in the PCOS population.

## 5. Conclusions

**Figure 3:**
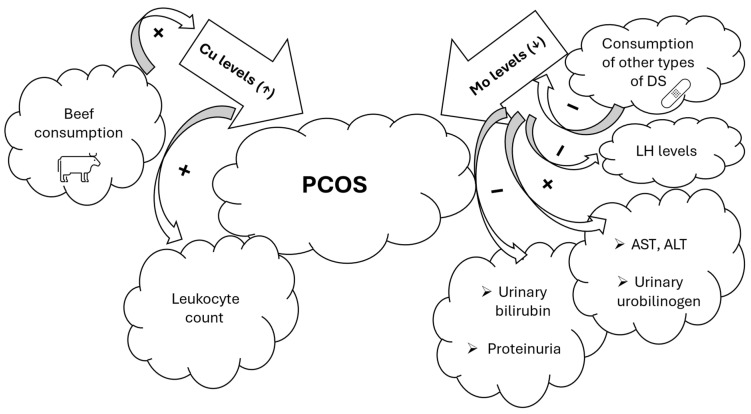
Summary of the main findings regarding associations of Cu and Mo levels with PCOS (statistical significance: *p* < 0.05). Legend: ALT—alanine aminotransferase; AST—aspartate aminotransferase; Cu—copper; DS—dietary supplement; LH—luteinizing hormone; Mo—molybdenum; PCOS—polycystic ovary syndrome.

In the present study, we found significantly higher serum Cu levels and significantly lower whole blood and serum Mo levels in women with PCOS compared to healthy controls. SG-normalized Mo levels appeared to be marginally significantly lower. Lifestyle, particularly dietary supplement consumption and diet, appeared to be important predictors of exposure to Cu (beef consumption) and Mo (consumption of cereals and boiled vegetables). Modifications in Cu and Mo levels can alter kidney and liver function and disrupt hormonal balance in PCOS women (Figure 3). However, these results still need to be interpreted with caution. Individual responses to TEs can vary and may be influenced by various factors such as genetics, environmental factors, and lifestyle interactions. In addition, the simultaneous exposure to multiple metals should always be taken into consideration. It is difficult to propose adequate dietary patterns and personalized lifestyle management strategies in the PCOS population, since this is the first study focusing on possible Mo interactions with PCOS pathophysiology. Further research is warranted to provide additional information on this relationship.

## Figures and Tables

**Table 1:** Sociodemographic characteristics of the study population.

		PCOS Group (*n* = 35)	Control Group (*n*= 35)	*p*-Value
Region of residence (%)	Osrednjeslovenska	82.9	77.1	0.630
	Gorenjska	0	2.9	
	Goriška	2.9	0	
	Jugovzhodna Slovenija	0	2.9	
	Obalno-Kraška	2.9	2.9	
	Podravska	8.6	2.9	
	Pomurska	2.9	2.9	
	Posavska	0	2.9	
	Primorsko-Notranjska	0	2.9	
	Savinjska	0	2.9	
Region of birth (%)	Osrednjeslovenska	54.3	62.9	0.804
	Gorenjska	5.7	8.6	
	Goriška	2.9	2.9	
	Jugovzhodna Slovenija	2.9	5.7	
	Koroška	2.9	0	
	Obalno-Kraška	2.9	2.9	
	Podravska	11.4	5.7	
	Pomurska	2.9	0	
	Posavska	0	2.9	
	Primorsko-Notranjska	0	2.9	
	Foreign country	14.3	5.8	
Level of education (%)	Primary school education	2.9	0	0.528
	Secondary school education	28.6	22.9	
	Tertiary education	5.7	0	
	Bachelor’s degree	31.4	45.7	
	Master’s degree	28.6	28.6	
	Doctorate	2.9	2.9	
Profession (%)	Lead position	0	2.9	0.109
	Expert/specialist	32.4	44.1	
	Technical job	11.8	14.7	
	Administrative job	20.6	5.9	
	Service sector	32.4	20.6	
	Agricultural sector	2.9	0	
	Ergonomically demanding	0	11.8	
Employment status (%)	Employed	68.6	62.9	0.519
	Unemployed	2.9	0	
	Student	28.6	34.3	
	Other	0	2.9	
Current workplace (%)	Lead position	0	3.1	0.180
	Expert/specialist	32.4	28.1	
	Technical job	11.8	9.4	
	Administrative job	20.6	43.8	
	Service sector	32.4	12.5	
	Agricultural sector	2.9	0	
	Oher	0	3.1	
Vicinity of manufacturing industry (%)	Yes	14.3	23.5	0.251
	No	85.7	76.5	
Vicinity of waste disposal site (%)	Yes	17.1	14.3	0.500
	No	82.9	85.7	
Home surroundings, agriculture (%)	Yes	34.3	29.4	0.430
	No	65.7	70.6	
Home surroundings, vegetable plots (%)	Yes	60.0	61.8	0.538
	No	40	38.2	
Home surroundings, fruit farming (%)	Yes	5.7	8.8	0.486
	No	94.3	91.2	
Environmental issue in the neighbourhood (%)	Yes	20.0	23.5	0.474
	No	80.0	76.5	
Metal implants, dental appliances (%)	Yes	25.7	40.0	0.154
	No	74.3	60.0	
Smoking status (%)	Smoker	20.0	23.5	0.474
	Former smoker	3.6	21.7	0.058
	Non-smoker, exposed to tobacco	35.7	38.5	0.529
Coffee consumption (%)	Yes	65.7	74.3	0.301
	No	34.3	25.7	
Alcohol consumption (%)	Never	14.3	5.7	0.472
	Occasionally	77.1	82.9	
	On regular basis	8.6	11.4	

Legend: PCOS—polycystic ovary syndrome. Pearson chi-squared test, Fisher’s exact test; statistical significance: *p* < 0.05.

**Table 2:** Statistically significant differences in anthropometric, ultrasonographic, and laboratory features in the PCOS and control groups of women (mean value ± standard deviation).

Clinical Feature (Mean ± SD)	PCOS Group (*n* = 35)	Control Group (*n* = 35)	*p*-Value
WC (cm)	15.3 ± 1.0 *	14.7 ± 0.79 **	0.027
BMI (kg/m^2^)	23.1 ± 2.79	21.3 ± 1.83	0.004
Menstrual cycle length (days)	45.3 ± 28.4	28.2 ± 1.56	<0.001
Endometrium thickness (mm)	5.57 ± 2.37	7.60 ± 2.96	0.004
AFC (average)	24.5 ± 10.2	10.1 ± 3.91	<0.001
Average OV (cm^3^)	10.1 ± 4.58	5.33 ± 1.70	<0.001
FSH (IU/L)	5.79 ± 2.08	7.58 ± 2.42	0.007
P (nmol/L)	6.01 ± 12.1	1.84 ± 0.87	0.018
TT (nmol/L)	1.31 ± 0.53	1.02 ± 0.29	0.006
FAI (%)	2.45 ± 1.37	1.50 ± 0.62	<0.001
A (nmol/L)	14.7 ± 6.50	10.3 ± 3.79	0.002
L (10^9^/L)	6.00 ± 1.34	5.45 ± 1.16	0.048

Legend: A—androstenedione; AFC—antral follicle count; BMI—body mass index; FAI—free androgen index; FSH—follicle-stimulating hormone; L—leukocyte count; OV—ovarian volume; P—progesterone; SD—standard deviation; TT—total testosterone; WC—wrist circumference. Mann–Whitney U test, statistical significance: *p* < 0.05; * *n* = 34, ** *n* = 30.

**Table 3:** Concentrations of TEs in urine in PCOS and control groups of women.

Biomarker (ng/g)	PCOS Group (*n* = 35)		Control Group (*n* = 35)		*p*-Value (SG-Norm)	Reference Value (ng/g) [38,44]
	GM (Min–Max); ORV (%)	SG-norm (Min–Max)	GM (Min–Max); ORV (%)	SG-norm (Min–Max)		
Mn	0.24 (<0.4 *–0.51)	0.24 (<0.4 *–0.50)	0.26 (<0.4 *–1.55)	0.26 (<0.4 *–0.54)	1.000 (0.599)	NA
Cu	11.1 (3.3–23.4); 0.00	11.0 (3.32–23.0)	11.2 (1.99–22.8); 0.00	11.1 (2.01–22.5)	0.542 (0.526)	1.48–29.6
Zn	372 (82–1787); 5.71	370 (81–1766)	425 (<40 *–1863); 14.3	421 (<40 *–1850)	0.470 (0.481)	49.4–1085
As	11.2 (2.59–155); 20.0	9.76 (0.64–140)	11.2 (2.59–154); 8.57	9.69 (0.64–138)	0.921 (0.902)	29.6
Se	31.2 (6.9–62.7); 34.3	31.1 (6.90–61.7)	34.5 (3.7–92.0); 45.7	34.2 (3.80–90.4)	0.112 (0.113)	1.97–39.4
Mo	47.7 (13.2–136); 0.00	47.5 (13.2–135)	63.8 (5.02–349); 5.71	63.3 (4.99–345)	0.050 (0.052)	168
Cd	0.30 (0.08–1.34); 2.86	0.30 (0.08–1.33)	0.33 (0.10–1.28); 11.4	0.32 (0.10–1.26)	0.664 (0.720)	0.69
Hg	0.84 (0.28–4.79); 5.71	0.84 (0.28–4.75)	0.74 (0.11–3.36); 0.00	0.73 (0.11–3.32)	0.769 (0.774)	3.95
Pb	0.57 (0.16–1.51); 11.4	0.57 (0.16–1.50)	0.51 (0.04–2.26); 14.3	0.51 (0.04–2.25)	0.700 (0.698)	1.09

Legend: GM—geometric mean; SG-norm—specific gravity normalization; Mn—manganese; Cu—copper; Zn—zinc; As—arsenic; Se—selenium; Mo—molybdenum; Cd—cadmium; Hg—mercury; NA—not available; ORV—outside the reference value; Pb—lead; PCOS—polycystic ovary syndrome. * Limit of detection; Mann–Whitney U test, statistical significance: *p* < 0.05.

**Table 4:** Concentrations of TEs in whole blood and serum in PCOS and control groups of women.

Biomarker (µg/L)	PCOS Group (*n* = 35)	Control Group (*n* = 35)	*p*-Value	Reference Interval (µg/L) ^a^
Whole blood				
	GM (min–max); ORV (%)	GM (min–max); ORV (%)		
Mn	8.41 (5.49–16.9); 0.00	8.59 (4.64–22.7); 2.86	0.720	4.0–19.0
Cu	867 (746–1160); 0.00	853 (673–1698); 2.86	0.382	550–1450
Zn	4683 (3546–6388); 26.0	4585 (3851–5625); 29.0	0.557	4000–7000
As	1.09 (0.43–8.26); 5.71	0.91 (0.33–33.9); 5.71	0.162	3.5
Se	156 (122–345); 11.4	154 (114–207); 14.3	0.760	80–170
Mo	0.60 (0.24–1.86); 2.90	0.71 (0.14–1.58); 2.90	0.024 *	<0.19–1.50
Cd	0.34 (0.11–1.64); 0.00	0.37 (0.13–3.81); 2.86	0.716	3.5
Hg	1.30 (0.51–6.58); 2.86	1.26 (0.12–11.1); 8.57	0.690	5.2
Pb	8.82 (4.45–17.9); 0.00	7.77 (3.18–21); 0.00	0.081	40.0
Serum				
Mn	0.55 (<0.31 ^#^–2.39); 34.3	0.47 (<0.31 ^#^–2.35); 48.6	0.180	<0.40–1.00
Cu	1028 (747–1443); 0.00	940 (751–1200); 0.00	0.026 *	600–1900
Zn	925 (688–1356); 2.86	877 (659–1600); 2.86	0.222	580–1350
As	0.45 (0.15–4.01); 14.3	0.36 (0.11–8.57); 5.71	0.137	1.4
Se	105 (85–138); 11.4	103 (83–125); 8.57	0.796	62–115
Mo	0.85 (0.31–2.35); 5.71	1.00 (0.17–2.57); 5.71	0.011 *	0.45–2.00
Cd	0.03 (<0.02 ^#^–0.31); 0.00	0.02 (<0.02 ^#^–0.44); 0.00	0.127	0.70
Hg	0.48 (0.15–1.88); 0.00	0.49 (0.14–3.20); 0.00	0.967	4.00
Pb	0.17 (<0.09 ^#^–2.12); 5.71	0.15 (0.05–2.17); 2.86	0.441	0.85

Legend: GM—geometric mean; Mn—manganese; Cu—copper; Zn—zinc; As—arsenic; Se—selenium; Mo—molybdenum; Cd—cadmium; Hg—mercury; ORV—outside the reference value; Pb—lead; PCOS—polycystic ovary syndrome. ^a^ Reference intervals for the Slovenian adult population [45]. ^#^ Limit of detection. * Statistically significant differences (Mann–Whitney U test, *p* < 0.05).

**Table 5:** The correlations of Mo and Cu levels with nutritional patterns in the PCOS (*n* = 35) and control groups of women (*n* = 35).

	Mo-SG		Mo-WB		Cu-S		Mo-S	
	r (*p*)							
	PCOS	Control	PCOS	Control	PCOS	Control	PCOS	Control
Consumption of other types of dietary supplements	−0.57 (0.020) *	0.18 (0.467)	−0.08 (0.762)	0.02 (0.948)	−0.369 (0.160)	−0.49 (0.039) *	−0.29 (0.281)	−0.09 (0.725)
Consumption of cereals	0.18 (0.303)	0.59 (<0.001) *	−0.03 (0.888)	0.61 (<0.001) *	−0.02 (0.889)	0.09 (0.624)	−0.02 (0.908)	0.48 (0.004) *
Consumption of pasta dishes	0.12 (0.505)	−0.02 (0.903)	0.14 (0.435)	−0.27 (0.110)	0.05 (0.771)	−0.35 (0.037) *	0.12 (0.474)	−0.24 (0.158)
Beef consumption	0.05 (0.782)	−0.14 (0.395)	0 (1)	−0.16 (0.350)	0.36 (0.032) *	0.08 (0.656)	0.16 (0.329)	−0.10 (0.571)
Canned fish consumption	0.02 (0.919)	0.16 (0.351)	−0.15 (0.386)	0.28 (0.099)	0.01 (0.959)	−0.13 (0.441)	−0.08 (0.611)	0.39 (0.019) *
Fresh vegetable consumption	0.21 (0.218)	0.42 (0.012) *	0.30 (0.071)	0.29 (0.093)	0.07 (0.709)	0.06 (0.719)	0.31 (0.066)	0.24 (0.160)
Boiled vegetable consumption	0.12 (0.493)	0.48 (0.003) *	0.11 (0.499)	0.40 (0.018) *	0.04 (0.804)	0.32 (0.055)	0.23 (0.166)	0.37 (0.029) *
Processed fruit consumption	0.26 (0.127)	0.13 (0.454)	−0.14 (0.409)	0.14 (0.418)	−0.16 (0.332)	−0.38 (0.024) *	−0.05 (0.748)	−0.05 (0.747)
Pickled vegetable consumption	−0.25 (0.13)	0.11 (0.496)	−0.28 (0.097)	0.01 (0.946)	0.09 (0.576)	−0.16 (0.336)	−0.36 (0.031) *	−0.04 (0.813)
Processed vegetable consumption	−0.40 (0.016) *	−0.14 (0.439)	−0.12 (0.488)	−0.14 (0.414)	0.04 (0.818)	−0.21 (0.209)	−0.40 (0.016) *	−0.15 (0.366)
Farinaceous food consumption	−0.06 (0.722)	0.51 (0.002) *	−0.22 (0.199)	0.11 (0.512)	0.13 (0.445)	0.04 (0.776)	−0.20 (0.260)	0.12 (0.480)
Tap water consumption	−0.14 (0.436)	0.17 (0.316)	0.20 (0.240)	0.28 (0.100)	−0.15 (0.381)	0.38 (0.026) *	0.10 (0.560)	0.30 (0.076)
Fruit juice consumption	−0.13 (0.450)	−0.25 (0.152)	−0.16 (0.332)	−0.43 (0.009) *	0.10 (0.548)	−0.28 (0.098)	−0.18 (0.312)	−0.55 (0.001) *
Black tea consumption	0.04 (0.797)	0.37 (0.029) *	0.02 (0.913)	0.05 (0.787)	−0.15 (0.360)	−0.21 (0.230)	0.02 (0.865)	0.09 (0.632)

Legend: Cu—copper; Mo—molybdenum; PCOS—polycystic ovary syndrome; S—serum; U—urine; WB—whole blood; SG—specific gravity; r—correlation coefficient. * Statistical significance: *p* < 0.05.

**Table 6:** Multiple linear regression and predictors of dietary exposure in study population.

		Estimate of Change (95% CI)		
		Total Population (*n* = 70)	PCOS Group (*n* = 35)	Control Group (*n* = 35)
**Dietary Habits**				
Mo-SG (ng/g)				
Consumption of other types of DSd ^a,b^	Once/month	-	-	-
	At least once/week	1.16 (0.92–1.46)	0.08 (0.57–1.14)	1.42 (1.05–1.93) *
	BMI adjustment	1.09 (0.86–1.39)	1.14 (0.82–1.60)	1.18 (0.86–1.61)
	Age adjustment	0.81 (0.65–1.03)	0.96 (0.68–1.36)	0.76 (0.55–1.03)
Consumption of cereals	Once/week	-	-	-
	2–6x/week	1.36 (1.11–1.66) *	1.24 (0.86–1.79)	1.43 (1.11–1.85) *
	Every day	1.68 (1.39–2.02) *	1.23 (0.87–1.75)	2.07 (1.68–2.56) *
	BMI adjustment	1.15 (0.94–1.41) *	1.16 (0.82–1.63)	1.14 (0.90–1.46) *
	Age adjustment	0.80 (0.66–0.97) *	0.86 (0.61–1.20)	0.79 (0.62–1.00) *
Consumption of boiled vegetables	Once/week	-	-	-
	2–6x/week	1.30 (1.00–1.67) *	1.36 (0.92–2.01)	1.37 (0.99–1.90)
	Every day	1.51 (1.18–1.92) *	1.15 (0.77–1.70)	2.12 (1.58–2.86) *
	BMI adjustment	1.08 (0.86–1.35)	1.10 (0.79–1.53)	0.90 (0.67–1.20)
	Age adjustment	0.83 (0.67–1.03)	0.85 (0.61–1.19)	0.81 (0.62–1.05)
Mo-WB (ng/g)				
Consumption of cereals	Once/week	-	-	-
	2–6x/week	1.36 (1.08–1.71) *	1.17 (0.80–1.70)	1.66 (1.26–2.18) *
	Every day	1.32 (1.05–1.65) *	0.94 (0.66–1.36)	1.80 (1.40–2.32) *
	BMI adjustment	0.99 (0.79–1.26)	1.06 (0.74–1.50)	0.86 (0.66–1.13)
	Age adjustment	0.92 (0.74–1.15)	0.98 (0.70–1.39)	0.90 (0.69–1.17)
Consumption of boiled vegetables	Once/week	-	-	-
	2–6x/week	1.27 (0.96–1.67)	1.04 (0.68–1.59)	1.60 (1.13–2.26) *
	Every day	1.30 (1.00–1.71)	1.08 (0.71–1.63)	1.84 (1.29–2.62) *
	BMI adjustment	0.95 (0.75–1.21)	1.02 (0.72–1.44)	0.78 (0.56–1.07)
	Age adjustment	0.94 (0.75–1.19)	1.00 (0.70–1.42)	0.92 (0.68–1.23)
Mo-S (ng/g)				
Consumption of cereals	Once/week	-	-	-
	2–6x/week	1.24 (0.99–1.57)	1.09 (0.75–1.60)	1.44 (1.07–1.94) *
	Every day	1.37 (1.09–1.71) *	0.99 (0.69–1.44)	1.80 (1.39–2.32) *
	BMI adjustment	1.01 (0.80–1.28)	1.07 (0.75–1.52)	0.91 (0.69–1.21)
	Age adjustment	0.85 (0.68–1.05)	0.85 (0.60–1.19)	0.87 (0.66–1.14)
Consumption of boiled vegetables	Once/week	-	-	-
	2–6x/week	1.29 (0.99–1.69)	1.27 (0.85–1.90)	1.39 (0.98–1.98)
	Every day	1.40 (1.08–1.81) *	1.21 (0.82–1.80)	1.91 (1.35–2.69) *
	BMI adjustment	0.97 (0.77–1.23)	1.05 (0.75–1.46)	0.76 (0.55–1.04)
	Age adjustment	0.86 (0.69–1.07)	0.82 (0.59–1.51)	0.88 (0.66–1.19)
Cu-S (ng/g)				
Consumption of beef	Less than once/month	-	-	-
	At least once/week	1.27 (1.03–1.57) *	1.51 (1.10–2.07) *	1.10 (0.79–1.54)
	BMI adjustment	1.21 (0.96–1.51)	1.27 (0.94–1.72)	1.10 (0.79–1.53)
	Age adjustment	1.10 (0.88–1.36)	1.14 (0.81–1.59)	1.23 (0.88–1.70)

Legend: CI—confidence interval; Cu—copper; DS—dietary supplement; Mo—molybdenum; PCOS—polycystic ovary syndrome; S—serum; U—urine; WB—whole blood; SG—specific gravity. * Statistical significance: *p* < 0.05; ^a^ *n* = 16 (PCOS group); ^b^ *n* = 18 (control group).

**Table 7:** The correlations of Mo and Cu levels with laboratory features in PCOS (*n* = 35) and a control group of women (*n* = 35).

	Mo-SG		Mo-WB		Cu-S		Mo-S	
	r (*p*)							
	PCOS	Control	PCOS	Control	PCOS	Control	PCOS	Control
TSH (mIU/L)	0.02 (0.916)	0.20 (0.260)	−0.01 (0.969)	0.56 (<0.001) *	0.05 (0.763)	−0.04 (0.811)	−0.04 (0.804)	0.39 (0.020) *
LH (IU/L)	−0.26 (0.119)	0.21 (0.222)	−0.24 (0.166)	0.19 (0.263)	−0.23 (0.188)	0.17 (0.322)	−0.35 (0.037) *	0.25 (0.137)
P (µg/L)	0.07 (0.709)	0.44 (0.008) *	−0.22 (0.200)	0.28 (0.108)	0.10 (0.576)	−0.06 (0.719)	−0.21 (0.235)	0.18 (0.296)
SHBG (nmol/L)	−0.09 (0.593)	−0.20 (0.248)	−0.06 (0.737)	−0.16 (0.371)	0.06 (0.747)	0.34 (0.044) *	0.01 (0.982)	0.01 (0.950)
Serum creatinine (µmol/L)	−0.05 (0.781)	0.10 (0.582)	−0.11 (0.522)	0.43 (0.008) *	0.13 (0.463)	0.15 (0.384)	0.09 (0.621)	0.51 (0.002)
AST (µkat/L)	0.17 (0.324)	0.09 (0.588)	0.34 (0.049) *	0.21 (0.231)	−0.06 (0.738)	0.11 (0.545)	0.53 (0.001) *	0.27 (0.113)
ALT (µkat/L)	0.10 (0.582)	−0.02 (0.928)	0.26 (0.133)	0.14 (0.435)	−0.06 (0.736)	−0.03 (0.847)	0.46 (0.005) *	0.16 (0.366)
L (10^9^/L)	0.00 (0.995)	−0.06 (0.731)	−0.02 (0.889)	0.15 (0.401)	0.40 (0.018) *	0.02 (0.925)	−0.12 (0.475)	0.04 (0.838)
MCV	0.38 (0.026) *	−0.08 (0.661)	−0.01 (0.948)	−0.34 (0.045) *	−0.08 (0.631)	−0.20 (0.258)	0.38 (0.024) *	−0.23 (0.189)
MCH	0.50 (0.002) *	−0.13 (0.467)	0.06 (0.742)	−0.36 (0.033) *	0.13 (0.463)	−0.19 (0.283)	0.34 (0.042) *	−0.22 (0.214)
U-SG	0.50 (0.002) *	0.54 (0.001) *	−0.42 (0.012) *	−0.01 (0.960)	0.18 (0.289)	0.03 (0.873)	−0.05 (0.789)	−0.05 (0.780)
U-pH	−0.03 (0.843)	−0.42 (0.013) *	0.20 (0.239)	−0.35 (0.037) *	−0.03 (0.884)	−0.30 (0.080)	0.02 (0.914)	−0.35 (0.038) *
U-prot.	−0.10 (0.587)	0.05 (0.760)	−0.37 (0.028)	0.06 (0.712)	0.30 (0.077)	−0.11 (0.522)	−0.32 (0.065)	0.10 (0.581)
U-urobil.	0.35 (0.041) *	0.15 (0.383)	−0.13 (0.464)	−0.20 (0.238)	−0.06 (0.729)	−0.19 (0.266)	−0.03 (0.847)	−0.15 (0.401)
U-bilirub.	−0.30 (0.075)	0.19 (0.283)	−0.34 (0.044) *	−0.20 (0.240)	0.08 (0.626)	−0.25 (0.140)	−0.37 (0.031) *	−0.27 (0.114)
U-creat. (mmol/L)	0.44 (0.009) *	0.64 (<0.001) *	−0.59 (<0.001) *	0.04 (0.826)	0.15 (0.379)	0.02 (0.895)	−0.24 (0.169)	−0.02 (0.926)

Legend: ALT—alanine aminotransferase; AST—aspartate aminotransferase; Cu—copper; LH—luteinizing hormone; L—leukocytes; MCV—mean corpuscular volume; MCH—mean corpuscular hemoglobin; Mo—molybdenum; P—progesterone; PCOS—polycystic ovary syndrome; r—correlation coefficient; S—serum; SHBG—sex hormone-binding globulin; TSH—thyroid-stimulating hormone; U—urine; U-bilirub.—urinary bilirubin; U-creat.—urinary creatinine; U-pH—urine pH; U-prot.—urinary proteins; U-SG—urine specific gravity; U-urobil.—urinary urobilinogen; WB—whole blood. * Statistical significance: *p* < 0.05.

## Data Availability

The data presented in this study are available on request from the corresponding author due to privacy and legal reasons.

## Supplementary Materials

The following supporting information can be downloaded at: https://www.mdpi.com/article/10.3390/metabo15020079/s1, Table S1. Differences in dietary patterns between the PCOS and control groups of women; Table S2. Differences in anthropometric, ultrasonographic, and laboratory characteristics between the PCOS and control groups of women (mean ± standard deviation); Table S3. The Zn/Cu ratios in urine, whole blood, and serum in the PCOS group (*n* = 35) and control group (*n* = 35) of women.

## Abbreviations

The following abbreviations are used in this manuscript:

A	Androstenedione
Alb	Albumin
Alpha-1-microgl.	Alpha-1-Microglobulin
ALT	Alanine Aminotransferase
As	Arsenic
AST	Aspartate Aminotransferase
AFC	Antral Follicle Count
BMI	Body Mass Index
CI	Confidence Interval
Cd	Cadmium
Cu	Copper
Creat	Creatinine
DHEAS	Dehydroepiandrosterone Sulphate
DS	Dietary Supplement
FAI	Free Androgen Index
FSH	Follicle-Stimulating Hormone
GM	Geometric Mean
GSH	Glutathione
HDL	High-Density Lipoprotein
Hg	Mercury
IgG	Immunoglobulin G
IR	Insulin Resistance
L	Leukocyte Count
LH	Luteinizing Hormone
LDL	Low-Density Lipoprotein
LOD	Limit of Detection
MCH	Mean Corpuscular Hemoglobin
MCHC	Mean Corpuscular Hemoglobin Concentration
MCV	Mean Corpuscular Volume
Mn	Manganese
Mo	Molybdenum
NA	Not Available
oGF	Glomerular Filtration Rate
ORV	Outside the Reference Value
OS	Oxidative Stress
OV	Ovarian Volume
P	Progesterone
Pb	Lead
PCOS	Polycystic Ovary Syndrome
PRL	Prolactin
r	Correlation Coefficient
RDW	Red Blood Cell Distribution Width
RV_95_	Reference Value (95th percentile)
S	Serum
SG	Specific Gravity
SG-norm	Specific Gravity Normalization
SHBG	Sex Hormone-Binding Globulin
Se	Selenium
SD	Standard Deviation
SOD	Superoxide Dismutase
TE	Trace Elements
TSH	Thyroid-Stimulating Hormone
TT	Total Testosterone
U	Urine
U-bilirub.	Urinary Bilirubin
U-creat.	Urinary Creatinine
U-Hb	Hemoglobin in Urine
U-ket.	Ketone Bodies in Urine
U-pH	Urine pH
U-urobil.	Urinary Urobilinogen
WB	Whole Blood
WC	Wrist Circumference
Zn	Zinc
γ-GT	Gamma-Glutamyl Transferase
25(OH)-vit.D	25-hydroxyvitamin D3
