# Supplementary material for: Association of Trace Elements with Polycystic Ovary Syndrome in Women—A Case-Control Study
Source: Metabolites. 2025 Jan 29;15(2):79. doi: 10.3390/metabo15020079 (PMC11857836; doi:10.3390/metabo15020079)
Supplement: Supplementary file 1 [file metabolites-15-00079-s001.zip › metabolites-3405940-supplementary.pdf]

Table S1. Differences in dietary patterns between the PCOS and control group of women.

Type of food and frequency of intake (x times/week ± SD)	PCOS (n=35)	Control (n=35)	p-value
**Mineral supplements**	3.67 ± 0.50[a]	3.24 ± 0.83[d]	0.175
**Vitamin supplements**	3.75 ± 0.62[b]	2.81 ± 1.05[c]	0.005
**Other types of dietary supplements**	3.87 ± 0.34[c]	2.94 ± 1.11[e]	0.001
**Bread**	3.86 ± 2.05	4.30 ± 2.26	0.282
**Cereals**	2.80 ± 2.22	2.64 ± 2.24	0.726
**Dairy products**	4.63 ± 2.40	5.23 ± 2.59	0.582
**Potatoes**	1.51 ± 0.92	1.76 ± 0.99	0.259
**Rice**	1.46 ± 1.09	1.60 ± 1.24	0.639
**Pasta dishes**	1.60 ± 0.95	2.13 ± 1.01	0.026
**Beef**	0.86 ± 0.73	0.97 ± 0.82	0.591
**Pork**	0.57 ± 0.66	0.66 ± 0.91*	0.995
**Chicken**	2.57 ± 2.00	1.93 ± 1.45	0.249
**Processed meat**	1.37 ± 1.37	1.34 ± 1.54	0.675
**Fresh fish**	0.89 ± 0.83	0.64 ± 0.56	0.219
**Canned fish**	0.83 ± 1.07	0.79 ± 0.89	0.945
**Seafood**	0.14 ± 0.36	0.19 ± 0.34	0.234
**Fresh vegetables**	4.26 ± 2.15	4.11 ± 2.26	0.695
**Boiled vegetables**	3.49 ± 2.39	3.54 ± 1.91	0.840
**Pickled vegetables**	1.14 ± 1.40	1.55 ± 1.59	0.241
**Fresh fruit**	5.66 ± 1.73	4.59 ± 2.29	0.017
**Cooked fruits**	0.31 ± 0.53	0.26 ± 0.59	0.745
**Processed fruits**	1.14 ± 1.31	1.17 ± 1.47	0.956
**Processed vegetables**	0.66 ± 1.03	0.60 ± 0.76	0.823
**Nuts**	2.46 ± 2.19	1.86 ± 1.66	0.301
**Farinaceous food**	0.97 ± 0.82	1.30 ± 1.35	0.406
**Desserts**	1.83 ± 1.58	1.89 ± 1.46	0.828
**Tap water**	6.80 ± 1.18	6.54 ± 1.56	0.321
**Mineral water (sparkling)**	0.77 ± 1.50	1.13 ± 1.53	0.305
**Still mineral water**	0.60 ± 1.33	1.24 ± 2.05	0.090
**Sweet carbonated drinks**	0.74 ± 1.58	0.71 ± 1.01	0.223
**Fruit juice**	1.37 ± 2.00	1.23 ± 1.18*	0.387
**Fruit tea**	1.97 ± 2.07	2.36 ± 2.12	0.358
**Black tea**	1.91 ± 2.48	0.82 ± 1.44*	0.071

Legend: PCOS–polycystic ovary syndrome; SD–standard deviation. Mann–Whitney U test, $P < 0.05$; [a] n=9; [b] n=12; [c] n=16; [d] n=17; [e] n=18; *n=34. Mann–Whitney U test, statistical significance $p < 0.05$.

Table S2. Differences in anthropometric, ultrasonographic, and laboratory characteristics between the PCOS and control group of women (mean ± standard deviation).

	PCOS (n=35)	Control (n=35)	p-value
**Anthropometric characteristics**			
WC (cm)	15.3 ± 1.0 *	14.7 ± 0.79**	0.027
BMI (kg/m2)	23.1 ± 2.79	21.3 ± 1.83	0.004
Menstrual cycle length (days)	45.3 ± 28.4	28.2 ± 1.56	< 0.001
**Ultrasonographic characteristics**			
Endometrium thickness (mm)	5.57 ± 2.37	7.60 ± 2.96	0.004
AFC (average)	24.5 ± 10.2	10.1 ± 3.91	< 0.001
Average OV (cm^3)	10.1 ± 4.58	5.33 ± 1.70	< 0.001
**Laboratory characteristics**			
TSH (mIU/l)	2.34 ± 0.82	2.43 ± 0.89	0.972
FSH (IU/l)	5.79 ± 2.07	7.58 ± 2.42	0.007
LH (IU/l)	7.37 ± 5.20	6.37 ± 2.71	0.801
PRL (µg/l)	11.9 ± 5.98	13.6 ± 5.84	0.182
17 β -estradiol (pmol/l)	243 ± 308	200 ± 93	0.518
Progesterone (nmol/l)	6.01 ± 12.1	1.84 ± 0.87	0.018
DHEAS (µmol/l)	6.81 ± 3.09*	6.15 ± 2.94	0.358
SHBG (nmol/l)	64.0 ± 30.4	78.6 ± 47.2	0.063

Total testosterone (nmol(l)	1.31 ± 0.53	1.02 ± 0.29	0.006
FAI (%)	2.45 ± 1.37	1.50 ± 0.62	<0.001
Androstenedione (nmol/l)	14.7 ± 6.50	10.3 ± 3.79	0.002
Serum creatinine (µmol/l)	65.8 ± 9.56	67.7 ± 9.02	0.466
oGF/1,73m^2 (ml/min)	89.1 ± 2.65	88.9 ± 4.09	0.576
AST (µkat/l)	0.39 ± 0.09	0.37 ± 0.06	0.768
ALT (µkat/l)	0.33 ± 0.15	0.32 ± 0.11	0.655
γ-GT (µkat/l)	0.25 ± 0.08	0.25 ± 0.10	0.364
Cholesterol (mmol/l)	4.49 ± 0.66	4.45 ± 0.85	0.617
HDL (mmol/l)	1.47 ± 0.31	1.43 ± 0.25	0.686
LDL (mmol/l)	2.82 ± 0.66	2.82 ± 0.81	0.629
Triglycerides (mmol/l)	0.73 ± 0.38	0.72 ± 0.27	0.558
Leukocyte count (10^9/l)	6.00 ± 1.34	5.45 ± 1.16	0.048
Red cell count (10^{12}/l)	4.47 ± 0.30	4.41 ± 0.29	0.814
Hemoglobin (g/l)	133 ± 8.70	130 ± 9.65	0.151
Hematocrit	0.40 ± 0.03	0.39 ± 0.02	0.466
Mean cell volume (fl)	89.5 ± 3.31	89.1 ± 4.11	0.879
Mean corpuscular hemoglobin (pg)	29.9 ± 1.28	29.5 ± 1.63	0.417
MCHC (g/l)	334 ± 9.79	331 ± 9.54	0.204
RDW (%)	12.4 ± 0.55	12.8 ± 1.17	0.094
Platelet concentration (10^9/l)	267 ± 54.4	251 ± 41	0.153
Mean platelet volume (fl)	10.5 ± 0.70	10.5 ± 0.80	0.939
U-SG	1.02 ± 0.00	1.02 ± 0.01	0.053
U-pH	5.94 ± 0.64	5.71 ± 0.47	0.131
U-prot.	0.17 ± 0.51	0.23 ± 0.65	0.724
U-ket.	0.20 ± 0.41	0.06 ± 0.24	0.076
U-urobil.	0.23 ± 0.43	0.37 ± 0.49	0.195
U-bilirub.	0.06 ± 0.24	0.03 ± 0.17	0.558
U-Hb	0.89 ± 1.18	1.40 ± 1.33	0.095
U-Lkci	0.49 ± 0.70	0.51 ± 0.78	0.929
U-IgG/creat. (g/mol)	0.22 ± 0.73	0.56 ± 2.31	0.335
U-alb./creat. (g/mol)	1.31 ± 2.99	2.63 ± 8.52	0.139
U-alpha-1-microgl./creat. (g/mol)	0.05 ± 0.14	0.11 ± 0.22	0.186
U-IgG/alb	0.10 ± 0.24	0.18 ± 0.53	0.357
U-creat. (mmol/l)	12.6 ± 5.41	15.0 ± 6.95	0.092
25-(OH)-vit.D (nmol/l)	70.4 ± 28.4*	60.6 ± 23.7	0.103

Legend: AFC–antral follicle count; Alb–albumin; alpha-1-microgl.–alpha-1-microglobulin; ALT–alanine aminotransferase; AST–aspartate aminotransferase; BMI–Body Mass Index; Creat–creatinine; DHEAS–dehydroepiandrosterone sulfate; FAI–Free Androgen Index; FSH–follicle-stimulating hormone; oGF–glomerular filtration rate; GGT–gamma-glutamyltransferase; HDL–high-density lipoprotein; IgG–imunoglobulin G; LDL–low-density lipoprotein; LH–luteinizing hormone; MCHC– mean corpuscular hemoglobin concentration; OV–ovarian volume; PCOS–polycystic ovary syndrome; PRL–prolactin; RDW–red blood cell distribution width; S–serum; SHBG–sex hormone-binding globulin; TSH– thyroid-stimulating hormone; U-bilirub.–urinary bilirubin; U-creat.–urinary creatinine; U-Hb–hemoglobin in urine; U-ket.–ketone bodies in urine; U-pH–urine pH; U-prot.–urinary proteins; U-SG–urine specific gravity; U-urobil.– urinary urobilinogen; WC–wrist circumference; 25(OH)-vit.D–25-hydroxyvitamin D3. Mann–Whitney U test, $P < 0.05$; *n = 34, **n = 30.

Table S3. Zn/Cu ratios in urine, whole blood and serum in PCOS group (n=35) and control group (n=35) of women.

**Zn/Cu ratio**		**GM (min–max)**	**p-value**
**Zn/Cu-SG**	PCOS group	33.5 (7.83–92.6)	0.541
	Control group	37.9 (10.7–125)	
**Zn/Cu-WB**	PCOS group	5.40 (3.88–8.28)	0.752
	Control group	5.37 (3.05–6.89)	
**Zn/Cu-S**	PCOS group	0.90 (0.58–1.54)	0.704
	Control group	0.93 (0.71–1.74)	

Legend: Cu–copper; GM–geometric mean; PCOS–polycystic ovary syndrome; S–serum; SG–specific gravity; WB–whole blood; Zn–zinc. Mann–Whitney U test, statistical significance $p < 0.05$.
